# Olive and grape seed extract prevents post-traumatic osteoarthritis damages and exhibits in vitro anti IL-1β activities before and after oral consumption

**DOI:** 10.1038/srep33527

**Published:** 2016-09-19

**Authors:** Elsa Mével, Christophe Merceron, Claire Vinatier, Stéphanie Krisa, Tristan Richard, Martial Masson, Julie Lesoeur, Vincent Hivernaud, Olivier Gauthier, Jérôme Abadie, Geoffroy Nourissat, Xavier Houard, Yohann Wittrant, Nelly Urban, Laurent Beck, Jérôme Guicheux

**Affiliations:** 1 INSERM, U791, LIOAD, Nantes, F-44042, France; 2 Union Grap’Sud, Cruviers-Lascours, F-30360, France; 3 Université de Nantes, UMR-S 791, LIOAD, UFR Odontologie, Nantes, F-44042, France; 4 Université de Bordeaux, ISVV, EA 3675 GESVAB, Villenave d’Ornon, F- 33883, France; 5 ONIRIS, UMR-S 791, LIOAD, Nantes, F-44307, France; 6 Cancers animaux, Modèle pour la recherche en Oncologie Comparée (AMaROC), Nantes, F-44307, France; 7 INSERM, UMRS 938, Metabolism and age-related joint diseases, Université Paris 6 Pierre & Marie Curie, Paris, F-75252 France; 8 Clermont Université, Unité de Nutrition Humaine, Clermont-Ferrand, F- 63122, France; 9 INRA, UMR 1019, UNH, CRNH Auvergne, Clermont-Ferrand, F- 63122, France; 10 CHU Nantes, PHU 4 OTONN, Nantes, F-44042, France

## Abstract

Polyphenols exert a large range of beneficial effects in the prevention of age-related diseases. We sought to determine whether an extract of olive and grape seed standardized according to hydroxytyrosol (HT) and procyanidins (PCy) content, exerts preventive anti-osteoathritic effects. To this aim, we evaluated whether the HT/PCy mix could (i) have *in vitro* anti-inflammatory and chondroprotective actions, (ii) exert anti-osteoarthritis effects in two post-traumatic animal models and (iii) retain its bioactivity after oral administration. Anti-inflammatory and chondroprotective actions of HT/PCy were tested on primary cultured rabbit chondrocytes stimulated by interleukin-1 beta (IL-1β). The results showed that HT/PCy exerts anti-inflammatory and chondroprotective actions *in vitro*. The preventive effect of HT/PCy association was assessed in two animal models of post-traumatic OA in mice and rabbits. Diet supplementation with HT/PCy significantly decreased the severity of post-traumatic osteoarthritis in two complementary mice and rabbit models. The bioavailability and bioactivity was evaluated following gavage with HT/PCy in rabbits. Regular metabolites from HT/PCy extract were found in sera from rabbits following oral intake. Finally, sera from rabbits force-fed with HT/PCy conserved anti-IL-1β effect, suggesting the bioactivity of this extract. To conclude, HT/PCy extract may be of clinical significance for the preventive treatment of osteoarthritis.

Osteoarthritis (OA) is a common inflammatory joint disease affecting a growing part of the elderly^1^ and is associated with a strong socio-economic impact^2^. On a pathophysiological point of view, OA is characterized by progressive cartilage loss, subchondral bone remodeling, osteophyte formation, and joint tissues inflammation^3^,^4^. Altered joint loading due to instability of the joint or ligament injury represents a significant risk factor for the onset and the progression of OA in humans^4^,^5^,^6^. From a molecular point of view, OA is a vicious cycle of inflammation and cartilage degradation. Abnormal mechanical loading causes an alteration in the metabolism of joint cells leading to a release of enzymes and inflammatory cytokines such as interleukin-1 beta (IL-1β). IL-1β then stimulates the synthesis of nitric oxide (NO), prostaglandin E_2_ (PGE_2_) and matrix metalloproteinases (MMPs)^7^,^8^. Among these proteases, MMP-13 is overexpressed in OA and mediates type II collagen and aggrecan breakdown which are the essential constituents of cartilaginous extracellular matrix (ECM)^9^. Fragments of aggrecan (*e.g.* NITEGE, VIPEN, DIPEN) and collagen are released from cartilage and induce the activation of synoviocytes and macrophages, resulting in paracrine secretion of cytokines and MMPs into the synovial fluid^10^,^11^. The released catabolic molecules are feeding the amplification loop of inflammation. For these reasons, inflammation and catabolism of ECM represent key events that can be targeted for the identification of novel therapeutic interventions in OA.

Despite recent advances in the understanding of the cellular and molecular processes involved in OA pathogenesis, curative treatments are still lacking. Pharmacological drugs currently available to treat OA patients only alleviate inflammation and pain but do not slow down, stop, or reverse the progression of the cartilage degradation and other tissue damages^12^. In this context, there is a growing interest in developing therapeutic strategies able to collectively blunt the progressive degradation of joint tissues while improving the relief of symptomatic inflammation-associated pain. Interestingly, numerous studies have recently highlighted the potential of natural compounds including nutraceuticals, to slow down or treat OA, thereby paving the way for new therapeutic approaches^13^,^14^,^15^,^16^,^17^,^18^,^19^,^20^,^21^,^22^.

Among these nutraceuticals, hydroxytyrosol (HT) is a bioactive phenolic compound mainly found in olive leaf and oil. HT is known for its powerful antioxidant and anti-inflammatory properties^23^,^24^. Previous studies have shown that oral intake of HT significantly decreased the *in vivo* acute inflammation triggered by LPS through the inhibition of PGE_2_ and NO production pathways^24^. Moreover, HT has the ability to relieve pain as described in a double-blind placebo-controlled trial conducted on 25 patients with knee OA^25^. Procyanidins (PCy) are active polyphenols found in many plants such as grape, pine bark, cocoa and raspberry. PCy exert numerous health promoting effects related to their antioxidant activity, but also to their ability to inhibit the synthesis of numerous inflammatory mediators^26^. Previous studies have thus shown that PCy from grape seed extract (GSPE) had the ability to alleviate inflammation *in vitro* and *in vivo* through the inhibition of NO, PGE_2_ and IL-1β production^27^,^28^. Interestingly, it has also been suggested that PCy may exert a protective effect on the ECM degradation as observed in OA through their targeted affinity with collagen^29^. A preventive effect of procyanidin B3 isoform on cartilage degradation has been reported recently in a murine model of OA^23^. Considered as a whole, these data raise the possibility that a combination of HT and PCy may exert complementary effects during the onset of OA. A mix of both extracts could be able to prevent both inflammation and joint tissue degeneration associated with disease. Despite this large body of data, some key aspects such as the fate of HT and PCy following oral administration still remains to be documented. As a matter of fact, the biological activities *in vivo* of polyphenol deeply depend on their bioavailability. In addition, after oral ingestion, polyphenols undergo several chemical modifications before reaching the bloodstream^22^. In this context, the evaluation of the bioavailability and the bioactivity are important parameters to be considered.

With respect to the large spectrum of OA symptoms, ranging from cartilage and bone alteration to synovial inflammation, the optimal treatment for OA would be a therapy that can not only blunt the progressive degradation of joint tissues but also improve the relief of inflammation-associated pain. In light of the promising data regarding the HT and PCy effects, we were interested in deciphering whether a combination of natural HT and PCy could simultaneously prevent inflammatory and catabolic effects related to the onset of OA. To address this issue, we used freshly isolated rabbit articular chondrocytes to test whether pre-treatment with HT/PCy extract mix could decrease the levels of IL-1β-induced inflammatory and catabolic markers. We also sought to determine the effect on the onset of disease of HT/PCy on two post-traumatic OA models in mouse and rabbit. Finally, we embarked on experiments to determine the bioavailability and bioactivity of HT/PCy extract mix and their metabolites in sera following force-fed gavages in rabbits.

## Materials and Methods

### Preparation of HT/PCy solution

Oleogrape^®^SEED, an extract from olive and grape seed (HT/PCy) was provided by Grap’Sud Union (Cruviers Lascours, France). It is composed of 6.5 mg HT/100 g and 30 mg PCy/100 g. HT/PCy extract was dissolved in high glucose Dulbecco’s Modified Eagle’s Medium (DMEM) (Thermo Fisher Scientific, Waltham-MA, USA) or in saline solution NaCl 0.9% (Sigma-Aldrich, Saint-Louis-MO, USA). Solutions were sterilized by filtration through 0.22 μm pore membranes.

### Ethical considerations in animal procedures

All animal procedures were approved by the institution’s animal welfare committee (CEEA Pays de la Loire. Agreement no. 02099.01) and were conducted in accordance with the European’s guidelines for the care and use of laboratory animals (2010/63/UE). Mice were housed in the animal facility of Nantes Faculty of Medicine (Agreement no. C44015). Rabbits were hosted in the animal facility of Nantes Faculty of Medicine or in Centre for Research and Preclinical Investigation (ONIRIS Agreement no. E44271).

### Experimental OA models

**Figure 1:**
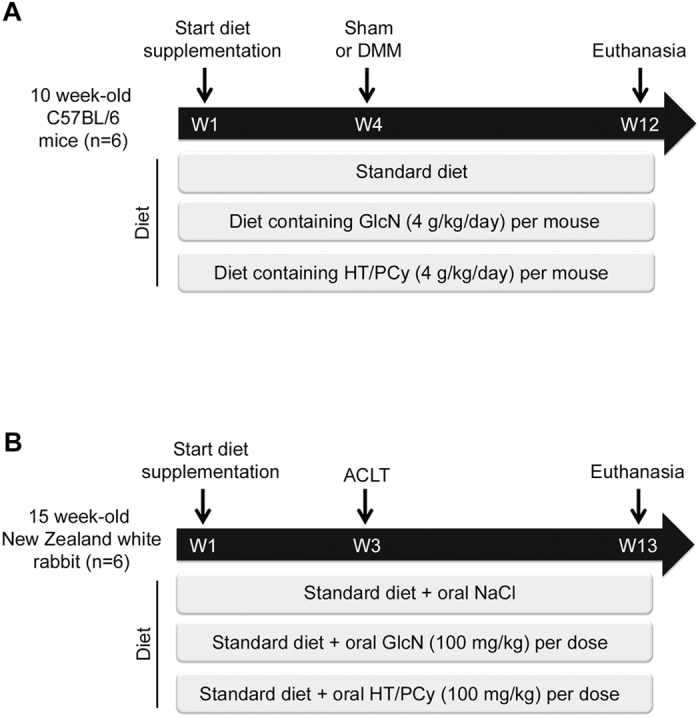
Experimental designs to evaluate the effectiveness of HT/PCy extract in animal models. (**A**) Mice (n = 6 per group) were bilaterally subjected to surgical DMM or sham operated. HT/PCy (4 g/kg) or GlcN (4 g/kg) were administrated daily by diet supplementation for 12 weeks starting 4 weeks before surgery. A third group was fed with standard diet (control group). Cartilage degradation was evaluated 8 weeks after DMM. (**B**) Rabbits (n = 6 per group) underwent anterior cruciate ligament transection (ACLT) of the right knee. Vehicle (NaCl 0.9%; negative control), glucosamine (GlcN) (100 mg/kg; positive control) or HT/PCy (100 mg/kg) was orally administrated every two days for 13 weeks starting 3 weeks before surgery. OA severity was evaluated at 10 weeks following surgery by radiographic and histological OARSI scoring.

Twenty-four 10 week-old male C57/BL6 mice were obtained from Janvier Labs (Saint Berthevin, France) and eighteen 15 week-old female New Zealand white rabbits were purchased from Hypharm-Grimaud (Roussay, France). All animals had reached full skeletal maturity at the time of the study. After one week of acclimatization, mice and rabbits were randomly assigned into 4 and 3 groups (n = 6 per group), respectively. In mice experiment, the sham group and the control group received a standard powder diet RM1 (Special Diet Services, Essex, UK) and experimental groups received either a standard diet enriched with D-(+)-glucosamine(GlcN) hydrochloride (Sigma-Aldrich) or with HT/PCy extract for 12 weeks. Based on previous *in vitro* and *in vivo* studies conducted in cartilage explants and ACLT rabbit model^24^,^30^, GlcN hydrochloride was used as positive control (Fig. 1A). Diet supplementation rates were calculated considering that mice consumed 4 g per day, which correspond to 4 g/kg/day of GlcN or HT/PCy extract. OA was experimentally induced in mice by bilateral destabilization of the medial meniscus (DMM) 4 weeks after the beginning of diet supplementation in order to assess the preventive effects of HT/PCy. Mice were euthanized 8 weeks after surgery by cervical dislocation (Fig. 1A). In rabbit experiment, the control group received a saline solution of NaCl 0.9% (Sigma-Aldrich) and experimental groups received GlcN (100 mg/kg) or HT/PCy (100 mg/kg) in 1 mL of saline solution every two days, administrated through gastric gavage for consecutive 13 weeks. Three weeks after the beginning of gavage, rabbits underwent a destabilization of the right joint induced by anterior cruciate ligament transection (ACLT). Rabbits were euthanized by an overdose of barbiturates 10 weeks after surgery (Fig. 1B). All joints were dissected for further histological examinations. One mouse in the control group was excluded from the analysis because the histological analysis revealed that the medial meniscotibial ligament had only been partially sectioned.

### Histological staining and OARSI scoring

After euthanasia, knee samples from each animal were fixed in 4% paraformaldehyde. Mouse and rabbit joints were decalcified using EDTA 0.5 M (pH 7.4) and DC3 solutions (VWR, Radnor, PA, USA) respectively. Dehydration was performed subsequently. Specimens were embedded in paraffin, and cut in 5 μm thick sections using microtome. Safranin O fast green and hematoxylin-eosin-safran (HES) stainings have been performed to visualize joint cells and matrices. Histological assessment was performed using the OsteoArthritis Research Society International (OARSI) scoring system by 2 well-versed observers in a blind random manner^31^,^32^. The severity of OA lesions was scored on a scale ranging from 0 to 5 using parameters such as chondrocyte death, hypertrophy, clusters, loss of Safranin-O staining, surface alteration, and bone modifications (total maximum score 25). The scoring system used is presented in Supplementary Table 1. Scoring was performed at different levels of the joint; at least two joint regions have been analyzed for each sample.

### Immunohistochemistry and image analysis

Immunohistochemistry was performed on deparaffinized and rehydrated sections with specific primary antibodies: type II collagen antibody (anti-mouse: 1:400, ab34712, Abcam and anti-rabbit: 1:100, 08631711, MP biomedicals), aggrecan antibody (anti-mouse: 1:100, ab1031, Merck Millipore and anti-rabbit: 1:100, MA3-16888, Thermo Fisher Scientific) and NITEGE antibody (anti-mouse: 1:500, PA1-1746, Thermo Fisher Scientific and anti-rabbit: 1:50, MBS442004, My Biosource (San Diego-CA, USA) for the detection of aggrecan cleavage. All sections were counterstained using Mayer’s hematoxylin (RAL Diagnostic, Martillac, France). Tissue staining was viewed using Nanozoomer 2.0 Hamamatsu slide scanner (Hamamatsu Photonics, Hamamatsu, Japan). Immunostaining intensity for NITEGE was quantified by determining the relative intensity of the stained articular cartilage matrix as previously described^33^. Briefly, images were converted into greyscale. The mean grey level values of 12 distinct regions of interest (50 × 50 pixels) from the femoral condyle and tibial plateau were determined. The value obtained was corrected by the mean of the gray levels of the extracellular matrix (10 × 10 pixels). Then, the corrected mean was subtracted from the blank (10 × 10 pixels). Finally, the value is expressed as fold increase over control condition. Measurements were performed using FIJI software.

### Radiographic imaging and OA score

Radiographic pictures of the rabbit’s hind limbs (antero-posterior and lateral views) were taken using a Convix 300 system (Picker International Inc, Cleveland, OH, USA). Pictures were taken following euthanasia to evaluate structural damage in the joints. Knee OA severity was quantitatively assessed using a radiographic score inspired by Kellgren & Lawrence^34^ and Boulocher *et al*.^35^. The scoring was performed by 2 independent blind readers. The severity of OA lesions was scored using multiple parameters such as calcification of menisci, tendons and ligaments, number and localization of osteophytes, bone modifications, and joint space narrowing. The scoring system used is summarized in Supplementary Table 2.

### Rabbit feeding and sera collection

Eight 15 week-old female New Zealand white rabbits were purchased from Hypharm-Grimaud. Animals were randomly divided into 2 groups (n = 4 per group). Control group was fed with saline solution (1 mL/kg) and experimental group received 6 doses of HT/PCy for 8 days. HT/PCy was administrated by gavage at the dose of 100 mg/kg (for cell culture) or 500 mg/kg (for UPLC-MS analysis) in saline solution as indicated in the figures legends. Venous blood was collected, centrifuged and sera were stored and frozen at −80 °C until their use in cell culture and Ultra Performance Liquid Chromatography-Mass Spectrometry (UPLC-MS) experiments.

### Cell culture

Rabbit articular chondrocytes (RAC) were harvested from tibial plateau and femoral condyles of New Zealand White rabbits as previously described^36^. Cells were plated at passage 1 in 96-well plates at a density of 100,000 cells/cm^2^ and maintained at 37 °C in a humidified atmosphere of 5% CO2 in control medium (10% FCS, 1% P/S). To analyze the preventive effects of native HT/PCy or their serum metabolites following digestion, cells were pre-incubated for 24 h in the presence of native HT/PCy at a concentration of 10 mg/L dissolved in DMEM supplemented with 5% FCS, 1% P/S or 2.5% of serum from force-fed rabbits in DMEM supplemented with 2.5% FCS, 1% P/S. Human recombinant IL-1β (1 ng/mL) (Millipore Corporation, Billerica-MA, USA) was then added for an additional 24 h.

### NO, PGE_2_ and MMP-13 quantification

Nitrate/Nitrite colorimetric assay and Prostaglandin E2 EIA kits were obtained from Cayman Chemical (Ann Arbor-MI, USA), Rabbit and Human ELISA Kits for MMP-13 detection were purchased from Cloud-Clone Corp (Houston-TX, USA) and Abcam^®^ (Cambridge, UK) respectively. The NO, PGE_2_ and MMP-13 levels measurements were performed according to manufacturer’s instructions.

### RNA extraction and real-time PCR

RNA was extracted using NucleoSpin® RNA II kit (Macherey-Nagel, Hoerdt, France) according to the manufacturer’s instructions. Reverse transcription was performed using SuperScript III Reverse Transcriptase (Thermo Fisher Scientific). Real-time polymerase chain reaction (PCR) was performed using Brilliant III^®^ SYBR^®^ Green Master Mix (Stratagene, La Jolla, CA, USA) on the Mx3000P^®^ QPCR System (Agilent Technologies, Santa Clara-CA, USA). PCR primers were synthesized by Eurofins DNA Genomics (Ebersberg, Germany) and sequences are reported in Supplementary Table 3. β-actin was used as reference gene and results were expressed as relative expression levels using the Livak method^37^.

### Extraction of phenolic compounds from serum

Sera (2.5 mL) from rabbit fed as described above were mixed with 7.5 mL of methanol for 2 min and ultrasonicated for 5 min. The mixture was centrifuged at 20,000 g for 10 min. The supernatant was collected and evaporated to dryness using a SpeedVac Concentrator. The dried extract was reconstituted in 200 μL of methanol/water (50:50 v/v). After centrifugation (20,000 g for 10 min), the supernatant was filtered through a PTFE 0.22 μm filter (Millipore Corporation) and stored at −80 °C until use for UPLC-MS analysis.

### Ultra-high performance liquid chromatography-mass spectrometry (UPLC-MS)

Phenolic compounds analyses were carried out using a 1290 Infinity UPLC from Agilent Technologies. The UPLC system was coupled to an Esquire 3000 plus mass spectrometer from Bruker Daltonics (Wissembourg, France). 5 μL were injected into a Zorbax SB-C18 column (2.1 × 100 mm, 1.8 μm) from Agilent Technologies. Two different solvents were used as a mobile phase: solvent A (water/formic acid 99.9:0.1 v/v) and solvent B (acetonitrile/formic acid 99.9:0.1 v/v), at a flow rate of 0.4 mL/min and a gradient as follows in solvent A: 0 min 1% B, 0.4 min 1% B, 2 min 10% B, 6 min 35% B.7 min 50% B, 8.8 min 70% B, 10.8 min 92% B, 11 min 100% B, 12 min 100% B, 12.2 min 1% B, 15.2 min 1% B. The MS/MS parameters were set as follows: negative mode; capillary tension +4000 V; nebulizer 40 psi; dry gas 10 L/min; dry temperature 365 °C; and scan range m/z 100 to 1400. Data were processed using HyStar 3.2 software (Bruker Daltonics).

### Statistical analysis

Each experiment was performed at least in triplicate. Results are expressed as mean ± SEM. Comparative studies were analyzed using an ANOVA test followed by post-hoc Fisher test with P values less than 0.05 considered as statistically significant.

## Results

### HT/PCy reduces IL-1β-induced levels of iNOS, COX2 and MMP13 transcripts and NO, PGE_2_ and MMP-13 production

**Figure 2:**
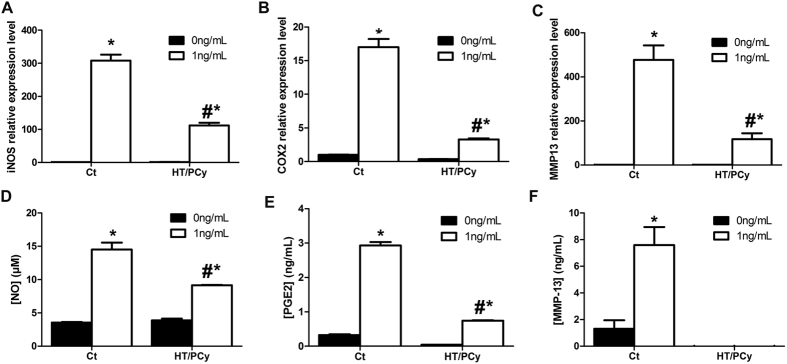
*In vitro* HT/PCy effects on iNOS, COX2 and MMP13 expression and NO, PGE_2_ and MMP-13 production. RAC were cultured for 24 h with HT/PCy and treated for an additional 24 h with IL-1β (1 ng/mL; white bar) or without IL-1β (corresponding to 0 ng/mL condition; black bar). The relative expressions of iNOS (**A**), COX2 (**B**) and MMP13 (**C**) compared to β-actin were evaluated by real time RT-PCR. NO production (**D**) was assessed using Greiss reaction, PGE_2_ (**E**) and MMP-13 (F) productions were measured using ELISA assay. ^#^p < 0.05 compared to the condition with IL-1β (1 ng/mL) alone, *p < 0.05 compared to the condition without IL-1β with the same extract solution.

We first evaluated whether HT/PCy treatment can modulate the expression levels of IL-1β-responsive genes involved in inflammation and catabolic processes in cartilage. RAC were pretreated with HT/PCy for 24 h then stimulated or not with IL-1β for 24 h. The expression of iNOS, COX2 and MMP13 transcripts were determined as well as associated products NO, PGE_2_ and MMP-13. As expected, results showed an increased expression of iNOS, COX2 and MMP13 in response to IL-1β treatment (Fig. 2). Interestingly, our results also demonstrated a significant decrease in IL-1β-induced iNOS, COX2 and MMP13 expression levels in HT/PCy condition (Fig. 2A–C). In addition to the gene expression levels, we monitored the synthesis of related products. Consistent with gene expression levels, the IL-1β-dependent NO, PGE_2_ and MMP-13 productions were also significantly decreased by the extracts in RAC (Fig. 2D–F). Of interest, HT/PCy totally suppressed the MMP-13 production triggered by IL-1β (Fig. 2F).These *in vitro* data demonstrated the anti-IL-1β effect of an HT/PCy pre-treatment in primary chondrocytes.

### Reduced severity of surgically-derivated OA following administration of HT/PCy in DMM mice

**Figure 3:**
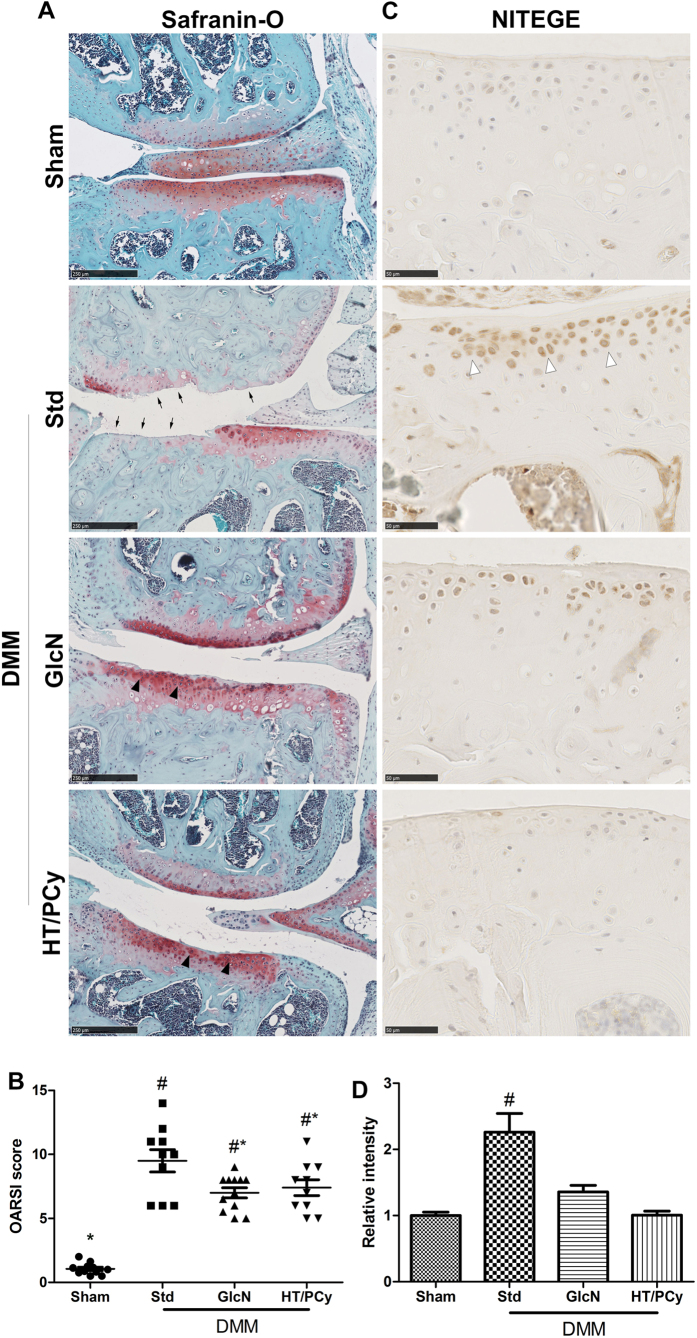
HT/PCy enriched diet reduces the severity of mid-stage OA in DMM mice. Mice (n = 6 per group) underwent sham surgery or DMM bilaterally. Mice received regular diet (Sham and Std groups) or diet supplemented with GlcN, HT/PCy for 12 weeks starting 4 weeks before surgery. Safranin O staining (**A**) and OARSI score (**B**) of sham or standard diet (Std), GlcN and HT/PCy diet groups at 8 weeks post-DMM. Immunohistochemical staining (brown signal) using antibody directed against NITEGE for the detection of aggrecan cleavage in sham or DMM mice receiving diet supplementation with GlcN, HT/PCy (**C**) and relative NITEGE staining intensity (**D**). *p < 0.05 compared to standard group, ^#^p < 0.05 compared to sham group. Bar represents 250 μm (**A**) and 50 μm (**C**).

To assess the safety of diet supplementation containing high dose of HT/PCy, the weight of the mice was monitored during 12 weeks. The initial and final body weights did not differ among the different experimental groups (data not shown) suggesting no adverse effects of HT/PCy. Safranin-O was used to evidence the glycosaminoglycan content from cartilage matrix. Sham-operated mice that received standard diet did not exhibit pathologic changes in articular cartilage (Fig. 3A). Eight-week post-DMM, sections of standard group reveals mid-stage OA which result in cartilage matrix delamination (black arrow) compared to sham group. In treated groups with GlcN or HT/PCy, the superficial zone was intact or presented only barely detectable superficial abrasion exhibiting safranin-O depletion. Moreover, some chondrocytes in treated groups were hypertrophic or organized in clusters demonstrating signs of early OA stage (black arrowhead; Fig. 3A). These histological observations were quantitatively confirmed using the OARSI scoring as shown in Fig. 3B. In standard group, OARSI score dramatically increased after DMM as compared to sham group. Of note, GlcN treated mice exhibited a significantly reduced OARSI score similar to that of HT/PCy treated mice.

To further investigate whether HT/PCy could protect the cartilage degradation subsequent to surgery, ECM components were analyzed by immunohistology. As expected, type 2 collagen expression was not affected among the different experimental groups (data not shown). Aggrecan staining intensity was strongly decreased after DMM in standard group compared to sham. Conversely, supplementing the diet with GlcN or HT/PCy did not result in a decrease in aggrecan immunostaining following surgery (Supplementary Figure 1). To confirm the absence of aggrecan degradation, we used NITEGE immunostaining corresponding to specific proteases having MMPs and aggrecanase activities. Sham-operated mice had no significant effect on the immunostaining for NITEGE-degradation product (Fig. 3C). The effects of surgery on aggrecan cleavage were confirmed by intense NITEGE expression in non-calcified cartilage and particularly in the pericellular region of standard group compared to sham group (Fig. 3C, white arrowhead), as previously described^33^. In contrast, immunohistochemistry showed that GlcN and HT/PCy treatments reduced the level of NITEGE in DMM mice compared to standard-treated mice (Fig. 3C). Surgery induced a 2.2 fold increased in the immunostaining intensity of NITEGE staining in standard diet treated mice. Quantifications of staining reveal that GlcN and HT/PCy dramatically reduced NITEGE immunostaining intensity to levels similar to that of sham operated mice (Fig. 3D). These data demonstrated that DMM surgery induced mid-stage OA lesions as compared to sham operated animals. Moreover, HT/PCy supplementation in DMM mice prevents from OA.

### Reduced severity of surgically-derivated OA following administration of HT/PCy in ACLT rabbits

**Figure 4:**
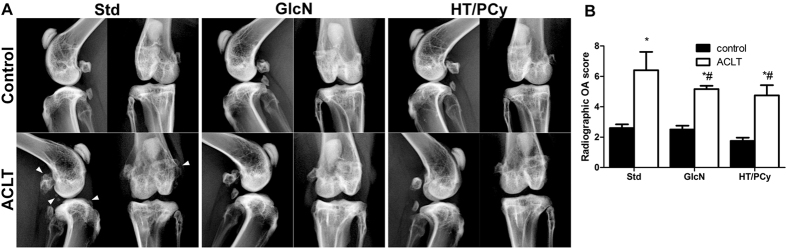
HT/PCy decreased radiographic OA score on ACLT rabbit model. Rabbits (n = 6 per group) underwent ACLT of the right knee. NaCl (Std), GlcN or HT/PCy were administrated every two days for 13 weeks starting 3 weeks before surgery. X-ray (**A**) and corresponding radiographic score (**B**) of non-operated knee (control) or operated knee (ACLT) untreated rabbits or treated with GlcN or HT/PCy at 10 weeks subsequence to surgery. *p > 0.05 compared to non-operated knee (control) within the same group; ^#^p > 0.05 compared to ACLT receiving NaCl (Std) group. Arrowheads show osteophytes formation after ACLT.

To strengthen the results obtained in a murine model of OA, we then evaluated the effects of diet supplementation with HT/PCy on a larger OA animal model. Taking into account that ACLT is closely related to the human clinical situation^38^, we examined the effects of HT/PCy enriched diet on OA severity induced by ACLT in rabbits. The severity of OA was firstly assessed by X-ray evaluation (Fig. 4). Non-operated knees (control) exhibit no joint alteration. In contrast, ACLT knees from rabbits fed with the standard diet (standard group) present OA pathological changes characterized by increased ligament calcification, osteophytes incidence and bone sclerosis (Fig. 4). Of interest, GlcN or HT/PCy intake decreased the lesion severity (Fig. 4A). Radiographic OA scoring reveals a significant decrease in treated groups compared to standard group. Our data also demonstrated that HT/PCy exerts similar effects on the X-Ray pathological OA changes when compared to GlcN (Fig. 4B).

**Figure 5:**
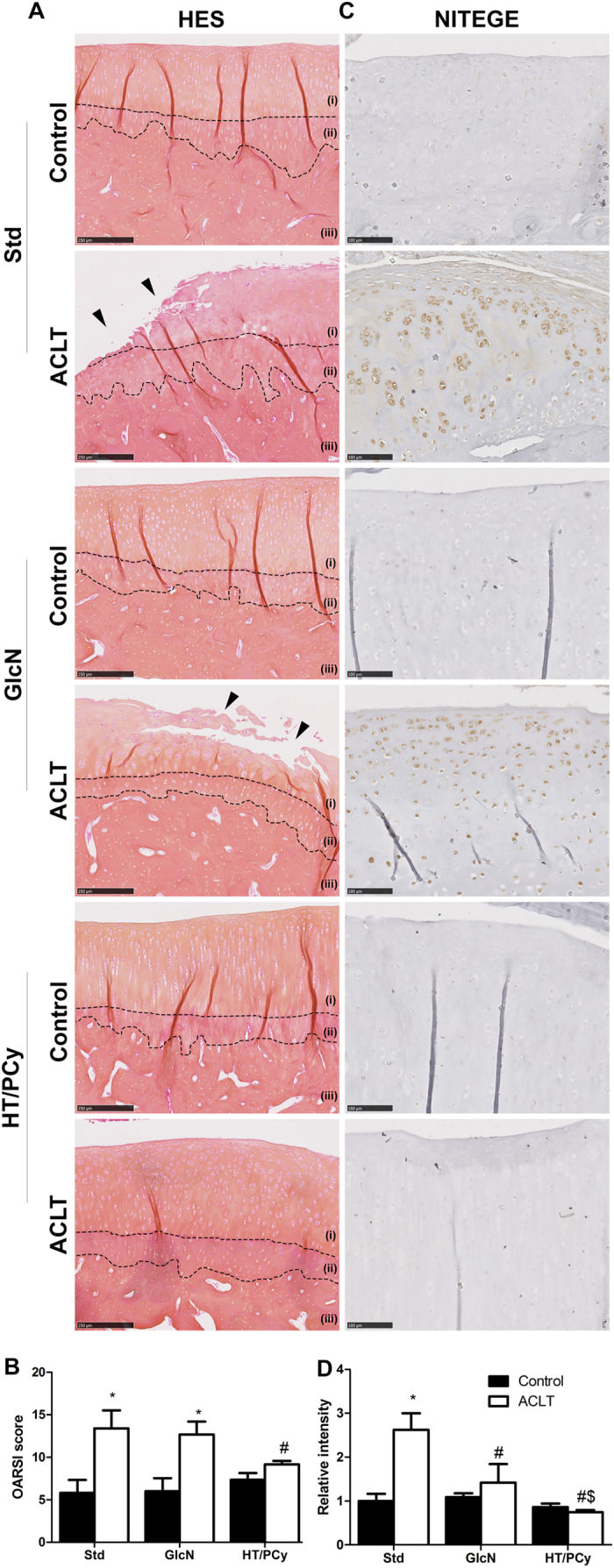
HT/PCy supplementation reduces the severity of mid-stage OA in ACLT rabbits. Rabbits (n = 6 per group) underwent ACLT of the right knee. NaCl (Std), GlcN or HT/PCy were orally administrated every two days for 13 weeks starting 3 weeks before surgery. HES staining of cartilage (**A**) and OARSI scores (**B**) of non-operated knee (control) or operated knee (ACLT) at 10 weeks post-surgery are shown. Immunohistochemical detection of aggrecan epitopes (NITEGE) (**C**) and relative staining intensity of the articular cartilage matrix (**D**) of standard diet (Std), GlcN and HT/PCy diet groups at 10 weeks after ACLT. (i) Non calcified-cartilage, (ii) calcified cartilage and (iii) sub-chondral bone. *p > 0.05 compared to non-operated knee (control) within the same group; ^#^p > 0.05 compared to ACLT receiving NaCl (Std) group, ^$^p > 0.05 compared to ACLT receiving GlcN. Bar represents 250 μm (**A**) and 100 μm (**C**).

To further investigate the *in vivo* effect of HT/PCy, we questioned whether HT/PCy intake may also impact cartilage alteration and subchondral bone changes at the onset of OA. Figure 5 shows that the articular cartilage surface of non-operated knees was smooth and that chondrocytes were organized in three appropriately oriented, well-organized zones. Cartilage erosion from the surface down to the deepest zone was observed for the group of rabbit that undergone ACLT and received standard diet corresponding to mid-stage OA. In experimental ACLT group treated with GlcN, the erosion was extended from the surface to the mid zone. Interestingly, ACLT rabbits treated with HT/PCy did not show any sign of cartilage erosion, but only presented cartilage swelling (œdema). GlcN was not found to exhibit a detectable effect on OARSI score in the ACLT rabbit model (Fig. 5B). Consistent with results obtained in mice, HT/PCy intake significantly decreased OARSI score severity in post-traumatic OA rabbit model (Fig. 5B).

To further decipher the effect of HT/PCy diet supplementation on cartilage matrix cleavage, type 2 collagen, aggrecan and NITEGE immunostainings were performed. As was observed in mice, the surgery did not affect type 2 collagen immunostaining in all groups (data not shown). On the contrary and as expected, surgery led to a substantial loss of aggrecan staining in comparison with non-operated knee. Interestingly, GlcN or HT/PCy intakes appear to prevent aggrecan degradation resulting from ACLT (Supplementary Figure 2). To specifically investigate the aggrecan cleavage, immunohistochemical detection of NITEGE epitope was performed. Non-operated knees among the different experimental groups had no significant immunostaining for NITEGE (Fig. 5C). However, ACLT led to intense NITEGE immunohistological detection in standard diet supplemented rabbits (standard). GlcN and HT/PCy gavages markedly reduced the level of aggrecan cleavage epitope induced by ACLT (Fig. 5C). Quantification of NITEGE intensity reveals that surgery strongly increased the intensity of NITEGE by 2.6-fold as compared to non-operated knee (control) in standard group. In addition, immunohistochemical detection showed that HT/PCy and GlcN did not affected NITEGE relative intensity in control knee compared to standard group. Interestingly, HT/PCy more drastically decreased the immunostaining intensity than GlcN (1.4-fold and 0.7-fold above non-operated knee (control) in standard group, respectively) (Fig. 5D).

Our results demonstrated that ACLT induced mid-stage OA, which causes pathological changes at X-ray and histological levels. Moreover, our results clearly confirmed, in a second model, the preventive effect of orally administrated HT/PCy on post-traumatic OA by analyzing the radiographic and histological changes. Interestingly, HT/PCy intake was found to be more potent than GlcN.

### Mass spectrometry identification of phenolic metabolites in rabbit serum

Since *in vivo* preventive effects of HT/PCy were observed following oral administration, we then questioned whether the sera of rabbits fed with HT/PCy may contain the regular metabolites of phenolic compounds. These metabolites were identified by UPLC-MS from the phenolic extract of HT/PCy and are reported in Table 1. All metabolites were picked out by the time retention peak T_R_ (min) and the transition of *m*/*z* [M–H]^−^. In serum of rabbits fed with HT/PCy, five metabolites from HT were found. Two HT derivatives were detected as hydroxytyrosol glucuronide and hydroxytyrosol sulfate, as reported in previous studies^39^,^40^. In addition, 2 tyrosol derivatives were also identified as tyrosol glucuronide and as tyrosol sulfate, as previously reported^41^. Finally, homovanillic acid sulfate was labeled^39^,^41^. Regarding rabbit’s metabolites derivating from PCy, nine metabolites were detected. Four catechin derivatives were identified. The peaks with the transitions were attributed to catechin glucuronide, catechin sulfate, methyl-catechin glucuronide and methyl-catechin sulfate, respectively^42^,^43^. Two epicatechin derivatives were attributed to epicatechin glucuronide and epicatechin sulfate, respectively^42^,^43^. In addition to proanthocyanidins, three other metabolites were detected as dihydroxyphenylvalerolactone sulfate^44^,^45^, dihydroxyphenylvaleric acid sulfate^44^, and hydroxyphenylpropionic acid^44^,^46^. None of these metabolites were found in the serum of rabbit force fed with NaCl. These results show the transformation of the extract by conjugation and its bioavailability in serum after oral intake.

### Sera from rabbits fed with HT/PCy reduces the IL-1β-induced levels of NO, PGE2 and MMP-13 production

**Figure 6:**
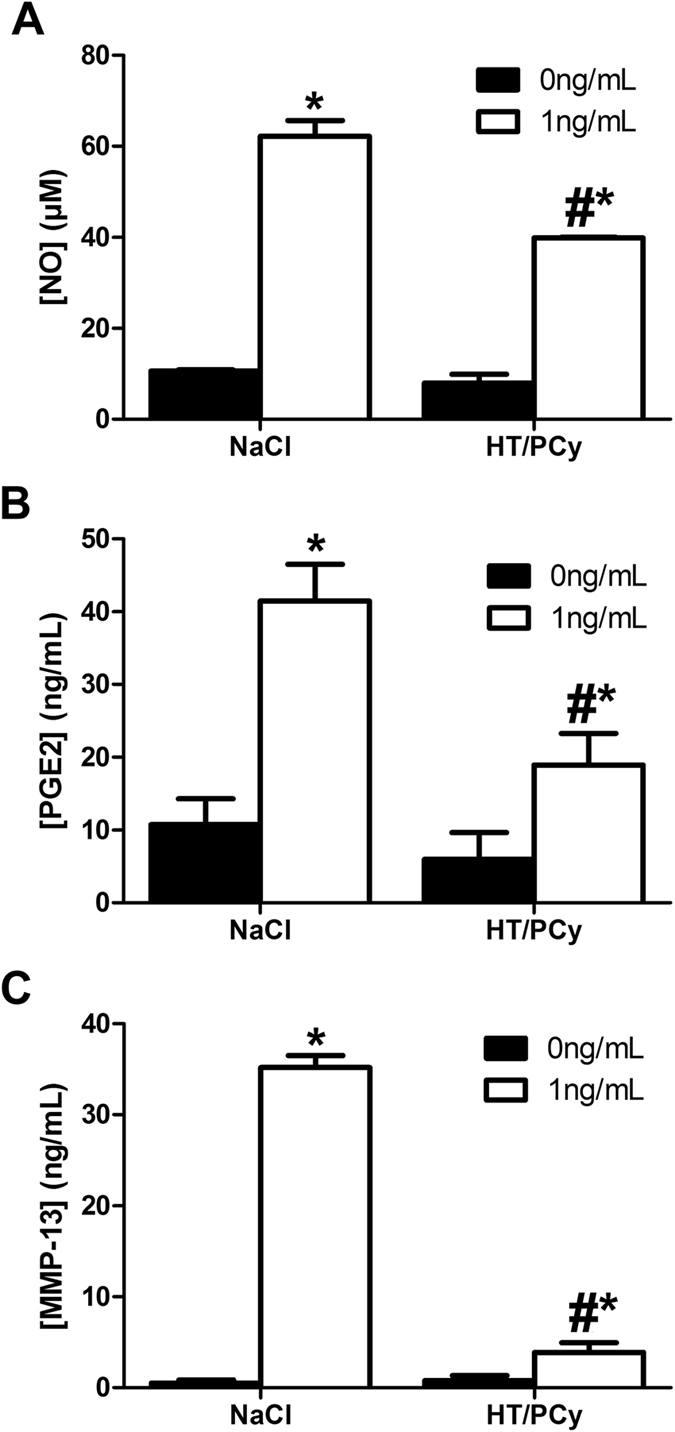
Effects of serum from rabbit fed with HT/PCy on the IL-1β-induced levels of NO, PGE_2_ and MMP-13 production. Rabbits received (n = 3 per condition) 6 doses of HT/PCy (100 mg/kg) or saline solution (NaCl) for 8 days and serum were harvested 2 h after the last gastric gavage. RAC were cultured during 24 h in presence of serum from rabbit at a final concentration of 2.5% (v/v) and stimulated with IL-1β (1 ng/mL) or its vehicle which was NaCl (0 ng/ml) for an additional 24 h. The NO (**A**) and PGE_2_ (**B**) and MMP-13 (**C**) released in culture media were measured. ^#^p < 0.05 compared to the NaCl condition with IL-1β (1 ng/mL), *p < 0.05 compared to the condition without IL-1β with the same extract solution.

To investigate the maintenance of the anti-IL-1β effects previously observed with native extract *in vitro*, we evaluated the anti-IL-1β effects of sera from rabbits after oral intake of HT/PCy. Results showed that serum from rabbit fed with HT/PCy extract down-regulated by 36% the NO production induced by IL-1β in RAC as compared to cells treated with serum from rabbit fed with saline (Fig. 6A). Similarly, serum from rabbit fed with HT/PCy extracts decreased by about 54% the PGE_2_ release triggered by IL-1β in RAC compared to control (Fig. 6B). Finally the serum from rabbit fed with HT/PCy extracts almost completely abolished the IL-1β-induced production of MMP-13 (Fig. 6C). Altogether, these results demonstrate that the sera of rabbit fed with HT/PCy contained metabolites with anti-IL-1β biological effects.

## Discussion

In this study, we demonstrated that olive and grape extracts containing HT/PCy exhibit preventive anti-IL-1β effects *in vitro* on isolated rabbit chondrocytes, and provided *in vivo* evidence in two animal models of post-traumatic OA that the combination of HT and PCy exerts preventive effects on the onset of OA. Importantly, we demonstrated that the sera of rabbits fed with phenolic-rich extracts exhibit *in vitro* anti-IL-1β activities in chondrocytes, thereby strongly suggesting that HT and PCy retain their biological activities in serum after oral consumption.

Despite that OA is the most common inflammatory and degenerative joint disease, no curative or preventive treatment has yet been clinically implemented^4^,^12^. In search of innovative therapeutic approaches, *in vitro* models recapitulating the inflammatory and degenerative processes are of high interest for drug screening. IL-1β is one of the pivotal cytokines involved in both degradative and inflammatory pathway associated to OA progression^47^,^48^. *In vitro*, IL-1β triggers the production of inflammatory markers including NO and PGE_2_ and proteolytic enzymes such as MMP-13 by chondrocytes^49^. Since IL-1β is a major cytokine involved in OA pathogenesis, *in vitro* models of IL-1β-treated chondrocytes have been widely used to screen drugs or natural compounds in the field of OA treatment^50^. In this context, we first embarked on experiments aiming at testing the effects of HT/PCy extract on the IL-1β-induced levels of NO, PGE_2_ and MMP-13 in rabbit primary chondrocytes. This study strongly suggests that a combination of HT and PCy may be potent to inhibit the inflammatory and catabolic effects of IL-1β. Interestingly, our data have shown that HT/PCy mix was able to prevent the effects of IL-1β by not only affecting the expression levels of transcripts encoding for iNOS, COX2 and MMP13, but also by reducing their inflammatory and catabolic related products NO, PGE_2_ and MMP-13 respectively. These data are consistent with two other independent studies establishing the chondroprotective effect of HT and PCy alone in H_2_O_2_-induced iNOS, COX2, MMP13 expression in chondrocytes^23^,^51^. Despite their promising aspect, one major limitation of this *in vitro* first set of data could be related to the use of monolayer culture model. It is thus well acknowledged that monolayer cultured chondrocytes rapidly lose their differentiated phenotype^36^. It would therefore be possible that isolated chondrocytes do not respond like chondrocytes embedded within their physiological microenvironment. Nevertheless, numerous studies have been conducted on cartilage explants or chondron culture and have consistently shown that IL-1β treatment triggered the production of similar inflammatory and degradative markers as in monolayer chondrocytes including NO, PGE2 and MMP-13^52^,^53^.

Despite recent advances in our understanding of the molecular mechanisms underlying OA pathogenesis^54^, pharmacological treatments only alleviate inflammation and pain but do not address the clinically relevant issue of joint tissue degeneration. In addition, medication often causes side effects including adverse liver and gastrointestinal reactions. In the search of a well-tolerated and orally deliverable medication able to prevent or treat OA early in the degenerative cascade, animal models that recapitulate most of the different aspects of human OA are a prerequisite to clinical translation. In addition to aging, traumatic injury remains one of the major causes of OA in human^55^,^56^. This has led us get interest into animal models in which OA can be induced by a reliable surgical intervention inducing a clinically relevant joint trauma such as meniscal lesion or cruciate ligament rupture. Among the *in vivo* models of post-traumatic OA, mouse is often the first-in-line model for its cost-efficiency, ease to handle and the availability of molecular tools^57^. In this context, we first used a model of mid-stage OA induced by DMM in mice. By using this murine model, we have undoubtedly demonstrated that a HT/PCy supplemented diet prior to surgery was able to significantly decrease post-traumatic OA severity as evidenced by reduced OARSI score and aggrecan-related NITEGE degradation product.

Although murine models of post-traumatic OA are instrumental for the preliminary screening of drugs, its relatively modest size as compared to human raise some issues. Notably, mice cartilage is extremely thin and it makes difficult to discern different layers^31^,^58^. This reduced thickness constitutes also an obstacle to histologically assess small defects^57^. Facing these limitations, and to strengthen the scientific relevance of our work, we were then interested in testing whether HT/PCy could also have preventing properties in a larger animal model that recapitulate one of the most frequent human traumatic joint injuries, the rupture of anterior cruciate ligament. Among the large animal model available, rabbit knees present the advantage of being quite similar, at least in gross appearance, to those of human^59^. By using, such an ACLT-induced OA model in rabbit, we have confirmed the preventive effects of HT/PCy on the severity of post-traumatic OA as evidenced by reduced X-Ray and OARSI scores as well as NITEGE detection.

Apart from their role in assessing the efficacy of drugs, animal models are also used to address the drug safety. In the field of OA research, various animal models such as murine, rabbit, canine, ovine are used to assess nutraceutical potency. As a consequence, a large variety of polyphenol derivative doses has been evaluated ranging from 0.1 mg/kg/day to 6 g/kg/day^22^. All animal models are not equivalent in terms of physiological and biochemical (*e.g.* absorption, distribution, metabolism, and excretion) parameters. Consequently, the tested dose and the effects produced by the concentration may vary greatly between two animal species. In this context, inter-species dose extrapolation, only based on body size, is likely to be irrelevant^60^,^61^,^62^,^63^,^64^. For experimental purpose, selected safe and effective therapeutic dose is however required. Considering these parameters and in the context of a safety assay in mouse model, the effects of HT/PCy were tested at relatively high dose (*i.e.* 4 g/kg/day). Our study demonstrated that HT/PCy was well tolerated at such a high dose and prevents OA from worsening. Based on this first set of data generated in mice and aiming at demonstrating the effects of HT/PCy in pharmacological condition, the dose was adjusted for application in the rabbit model. Referring to metabolic body size adjustment formula outlined in literature, rabbits received HT/PCy mid-range doses of 100 mg/kg/day^60^,^61^,^62^,^63^,^64^, which are, according to metabolic body size, a 10 times lower dose than those given to mice. Despite this discrepancy and consistently with the results obtained in mice, we found that HT/PCy used at mid-range dose in rabbits has preventive effects on OA severity.

It is nowadays commonly accepted that OA is a heterogeneous disease described with five different phenotypes: post-traumatic, pain, ageing, metabolic and genetic^4^,^65^. In the light of these considerations, two different approaches could be considered. On the first hand, strategies that consist in targeting a specific OA phenotype could be implemented. On the other hand, a second approach that aims at developing treatments with broad spectra could also be contemplated. With respect to the promising ability of HT/PCy to counteract *in vitro* IL-1β-dependent inflammatory and catabolic events as well as OA severity in post-traumatic OA models, it could be interesting to further explore the anti-OA effects of HT/PCy mix in other OA models mimicking the human metabolic, ageing, pain and genetic phenotypes.

In the perspective of a transfer from bench to bedside, the bioavailability, *i.e*. ability to reach blood flow, of natural anti-osteoarthritic molecules after oral administration is a key pre-requisite. Surprisingly and despite a huge number of studies dealing with the anti-osteoarthritic properties of nutraceuticals^66^, no study has directly addressed the bioavailability of phenolic compound metabolites in the blood stream after ingestion. The systemic bioavailability depends on the various chemical modifications that compounds undergo after absorption from the intestine barrier (*e.g.* deglycosylation, glucuronate modification, and/or conjugation with sulfates) that lead to the formation of metabolites chemically different from the native compound^67^,^68^. The chemical modification can alter or inactivate biological activity of molecules. This was well illustrated by Yang *et al*., who reported that hesperitin, a metabolite of hesperidin (a polyphenol found in citrus fruits), contained in serum of hesperidin-fed rats, showed potent anti-inflammatory activity, whereas the native hesperidin molecule did not^69^. On the contrary, it cannot be excluded that the bioactivity of native molecule can be suppressed by metabolic modifications following ingestion. We therefore sought to determine whether the serum of rabbit fed with HT/PCy contains bioactive metabolites of phenolic compounds. To address this issue, two complementary methods have been used. The first direct method consists of identifying the presence of specific and already known metabolites of phenolic compounds by UPLC-MS in collected serum. Accordingly, the sera of rabbits force-fed with HT/PCy were found to contain 5 metabolites of HT and 9 of PCy. It would be of interest to assess HT/PCy bioavailability in synovial fluid after oral administration. Unfortunately, joint fluid volume in normal 15 week-old New Zealand white rabbits is unsatisfying to be collected and analyzed by UPLC-MS. The second indirect method consists of testing the biological activity of sera from rabbits force-fed with extracts. In this purpose, sera collected from rabbits force-fed with extracts were found to exhibit an anti-IL-1β effect in *in vitro* models of cultured chondrocytes. Of interest, our data clearly indicate that the sera of rabbits fed with HT/PCy extract significantly down-regulated the IL-1β-induced levels of NO, PGE_2_ and MMP-13. Considered together, these results strongly suggest that HT and PCy conserved their bioactivities in serum after undergoing digestive process in rabbit. It remains however difficult to totally rule out the possibility that HT/PCy oral intake may trigger the production of yet unidentified secondary mediators in sera that also have anti-IL-1β effect. No study, to our knowledge, has addressed this interesting assumption that should deserve further consideration.

To conclude, HT/PCy exerts *in vitro* anti-IL-1β effects in chondrocytes and significantly reduces the severity of post-traumatic OA in mice and rabbit models. The sera of rabbits force fed with HT/PCy was found to contain the regular digestive metabolites of polyphenols and to exhibit *in vitro* anti-IL-1β effects in chondrocytes thereby suggesting that HT/PCy are bioavailable and bioactive after oral intake.

## Additional Information

**How to cite this article**: Mével, E. *et al*. Olive and grape seed extract prevents post-traumatic osteoarthritis damages and exhibits in vitro anti-IL-1β activities before and after oral consumption. *Sci. Rep.* **6**, 33527; doi: 10.1038/srep33527 (2016).

## Supplementary Material

Supplementary Information

## Figures and Tables

**Table 1:** Mass spectrometry identification of phenolic metabolites in rabbit serum.

Compounds	T_R_ (min)	[M–H]^−^	MS/MS
***HT***			
hydroxytyrosol glucuronide	3.0	329	153
hydroxytyrosol sulfate	3.1	233	153
homovanillic acid sulfate	3.6	261	181
tyrosol glucuronide	5.5	313	137
tyrosol sulfate	5.7	217	137
***PCy***			
catechin glucuronide	3.5	465	289
epicatechin glucuronide	3.8	465	289
catechin sulfate	4.0	369	289
methyl catechin glucuronide	4.2	479	303
epicatechin sulfate	4.4	369	289
dihydroxyphenylvalerolactone sulfate	4.5	287	207
methyl catechin sulfate	4.6	383	303
dihydroxyphenylvaleric acid sulfate	5.1	289	209
hydroxyphenylpropionic acid	5.2	165	121

Rabbits received 6 doses of HT/PCy (500 mg/mL) for 8 days, 2 hours after the last dose the serum was harvested. The characterization of the metabolites in the rabbit serum was based on their ion fragmentation in the MS and MS/MS modes. Metabolites were identified by the time retention (T_R_) (min) and the transition of *m*/*z* [M–H]^−^. Mass spectral characteristic of phenol metabolites in serum from rabbits fed a fraction HT/PCy.
