# Supplementary material for: Olive and grape seed extract prevents post-traumatic osteoarthritis damages and exhibits in vitro anti IL-1β activities before and after oral consumption
Source: Sci Rep. 2016 Sep 19;6:33527. doi: 10.1038/srep33527 (PMC5027597; doi:10.1038/srep33527)
Supplement: Supplementary Information [file srep33527-s1.pdf]

Supplementary Figure 1

Olive and grape seed extract prevents post-traumatic osteoarthritis damages and exhibits in vitro anti IL-1β activities before and after oral consumption

E. Mével, C. Merceron, C. Vinatier, S. Krisa, T. Richard, M. Masson, J. Lesoeur, V. Hivernaud, O. Gauthier, J. Abadie, G. Nourissat, X. Houard, Y. Wittrant, N. Urban, L. Beck, J. Guicheux

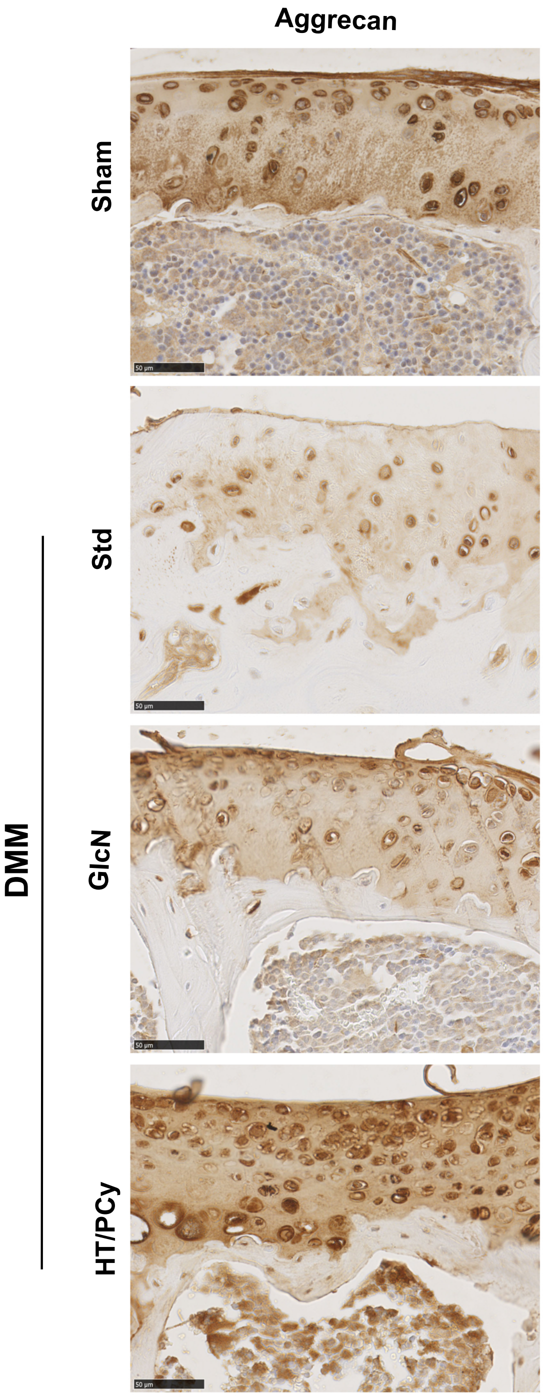

Supplementary Figure 1:
Mice (n=6 per group) underwent sham surgery or DMM bilaterally. Mice received regular diet (Sham and Std groups) or diet supplemented with GlcN, HT/PCy for 12 weeks starting 4 weeks before surgery. Immunohistochemical staining (brown signal) using antibody directed against aggrecan in sham or DMM mice receiving diet supplementation with GlcN, HT/PCy. Bar represents 50µm.

Olive and grape seed extract prevents post-traumatic osteoarthritis damages and exhibits in vitro anti IL-1β activities before and after oral consumption

E. Mével, C. Merceron, C. Vinatier, S. Krisa, T. Richard, M. Masson, J. Lesoeur, V. Hivernaud, O. Gauthier, J. Abadie, G. Nourissat, X. Houard, Y. Wittrant, N. Urban, L. Beck, J. Guicheux

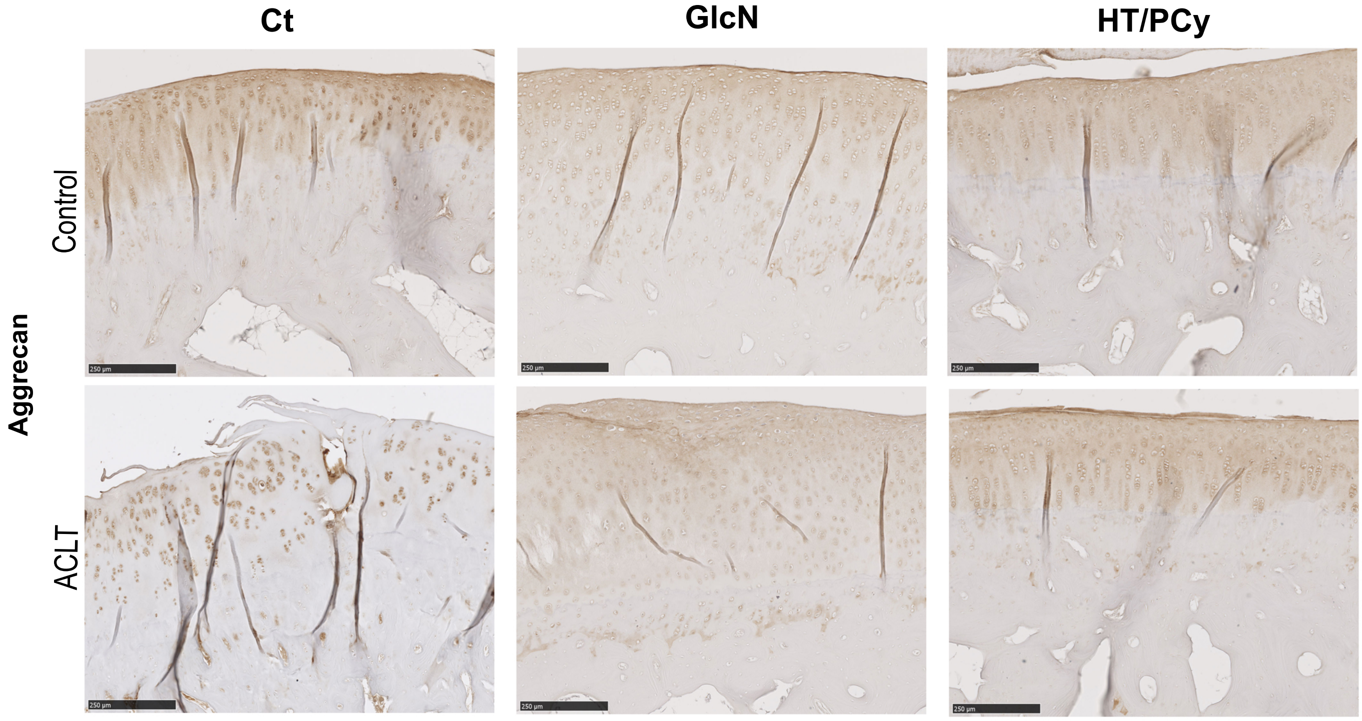

Supplementary Figure 2:
Rabbits (n=6 per group) underwent ACLT of the right knee. NaCl (Std), GlcN or HT/PCy were orally administrated every two days for 13 weeks starting 3 weeks before surgery. Immunohistochemical detection of aggrecan of the articular cartilage matrix of standard diet (Std), GlcN and HT/PCy diet groups at 10 weeks after ACLT. Bar represents 250µm.

Olive and grape seed extract prevents post-traumatic osteoarthritis damages and exhibits in vitro anti IL-1β activities before and after oral consumption

E. Mével, C. Merceron, C. Vinatier, S. Krisa, T. Richard, M. Masson, J. Lesoeur, V. Hivernaud, O. Gauthier, J. Abadie, G. Nourissat, X. Houard, Y. Wittrant, N. Urban, L. Beck, J. Guicheux

Scoring category	Observation	Score
Chondrocytes death	None	0
	0-10%	1
	10-25%	2
	25-50%	3
	50-75%	4
	75-100%	5
Hypertrophy	None	0
	Presence	1
Clusters	None	0
	Superficial zone	1
	Middle zone	2
	Complexe structures	3
	Mild layer cyst formation	4
Loss of Safranin-O staining	None	0
	Superficial zone of non-calcified cartilage	1
	Middle zone of non-calcified cartilage	2
	Non-calcified cartilage and intense staining around clusters	3
	Total with staining around clusters	4
	Total	5
Surface	Intact	0
	Superficial fibrillation or abrasion	1
	Deep fibrillation	2
	Vertical fissures	3
	Delamination/ excavation	4
Bone	None	0
	Denudation	4
	Microfracture	5
	Remodeling	6
	**Total**	**25**

Supplementary Table 1: The OARSI scoring system used to evaluate OA severity adapted from Glasson, S.S., *et al.*, 2010 and Pritzker, K.P., *et al.*, 2005.

Olive and grape seed extract prevents post-traumatic osteoarthritis damages and exhibits in vitro anti IL-1β activities before and after oral consumption

E. Mével, C. Merceron, C. Vinatier, S. Krisa, T. Richard, M. Masson, J. Lesoeur, V. Hivernaud, O. Gauthier, J. Abadie, G. Nourissat, X. Houard, Y. Wittrant, N. Urban, L. Beck, J. Guicheux

	Scoring category	Observation	Score
	Menisci calcification	None<br>1<br>2	0<br>1<br>2
Number of visible osteophytes	Medial tibial condyle	None<br>Low - Moderate<br>Moderate - Severe	0<br>1<br>2
	Medial femoral condyle	None<br>Low - Moderate<br>Moderate - Severe	0<br>1<br>2
	Medial fabella	Absence<br>Presence	0<br>1
	Structural modifications of subchondral bone (sclerosis)	None<br>Low - Moderate<br>Moderate - High	0<br>1<br>2
	Width of the joint space	Normal<br>Reduced<br>Absent	0<br>1<br>2
	Calcification of tendons and ligaments	None<br>1 site<br>> 1 site	0<br>1<br>2
		Total	13

Supplementary Table 2: The radiographic scoring system used to evaluate OA severity inspired by Kellgren, J.H. and J.S. Lawrence, 1957 and and Boulocher C.B., *et al*., 2010.

Olive and grape seed extract prevents post-traumatic osteoarthritis damages and exhibits in vitro anti IL-1β activities before and after oral consumption

E. Mével, C. Merceron, C. Vinatier, S. Krisa, T. Richard, M. Masson, J. Lesoeur, V. Hivernaud, O. Gauthier, J. Abadie, G. Nourissat, X. Houard, Y. Wittrant, N. Urban, L. Beck, J. Guicheux

Species	Amplified gene	Primer sequences (5'-3')	Gene Bank Reference	Amplicon Size (bp)
Rabbit	iNOS	FP : TGACGTCCAGCGCTACAATA<br>RP : TGCGTCTCCAGTCCCATC	NM_000625.4	60
	COX2	FP : GGAAGCGCTCTACGGCGACA<br>RP : CCCCAAAGATGGCATCCGGGC	NM_001082388.1	86
	MMP13	FP : TTTTGAAGACACGGGCAAG<br>RP : TCATCATAGCTCCAGACTTGGTT	NM_002427.2	60
	ACTB	FP : CCCATCTACGAGGGCTACGC<br>RP : TCCTTGATGTCCCGCACGATC	NM_001101683.1	152

Supplementary Table 3: Amplified gene, sequences of primers and gene bank accession reference used for real-time PCR analysis and size of PCR products. iNOS : inducible nitric oxide synthase ; COX2 : cyclo-oxygenase; MMP-13: matrix metallopeptidase 13; ACTB: β-actin; FP: forward primer; RP: reverse primer.
